# Robotic single-site versus laparoendoscopic single-site hysterectomy: a propensity score matching study

**DOI:** 10.1007/s00464-015-4292-9

**Published:** 2015-06-20

**Authors:** Jiheum Paek, Jung-Dong Lee, Tae Wook Kong, Suk-Joon Chang, Hee-Sug Ryu

**Affiliations:** Department of Obstetrics and Gynecology, Ajou University School of Medicine, 164 World Cup-ro, Yeongtong-gu, Suwon, 443-380 Korea; Office of Biostatistics, Ajou University School of Medicine, Suwon, Korea

**Keywords:** Robotics, Hysterectomy, Laparoscopy

## Abstract

**Background:**

The aim of this study was to compare the surgical outcomes of robotic single-site (RSS-H) and laparoendoscopic single-site total hysterectomy (LESS-H) and to evaluate the feasibility of RSS-H in patients with benign gynecologic disease.

**Methods:**

The RSS-H was performed using the da Vinci single-site surgical platform, and the LESS-H using a single multi-channel port system at the umbilicus. Among 467 consecutive patients who had undergone total hysterectomy for benign gynecologic disease, surgical outcomes were compared between RSS-H group (*n* = 25) and LESS-H group (*n* = 442) after propensity score matching.

**Results:**

All operations were completed robotically and laparoscopically without conversion to laparotomy, respectively. The RSS-H group had longer operating times and less operative bleeding compared to the LESS-H group. While the LESS-H showed 1.4 % of major complication rate, the RSS-H had no perioperative complication. Even after propensity score matching, the RSS-H still showed longer operating times (170.9 vs 94.1 min, *p* < 0.0001) and less operative bleeding (median estimated blood loss, 20 vs 50 ml, *p* = 0.009; mean hemoglobin drop, 1.6 vs 2.0 g/dl, *p* = 0.038) than the LESS-H.

**Conclusions:**

The RSS-H could be a feasible and safe procedure in appropriately selected patients with benign gynecologic disease, and further experience and technical refinements will continue to improve operative results. Prospective randomized trials will permit the evaluation of the potential benefits of the RSS surgery as a minimally invasive surgical approach.

In the gynecologic field, laparoendoscopic single-site (LESS) surgery can be performed widely to meet female patients’ demands in which they would like to have less surgical scarring [1–4]. However, although a lot of studies have been showed regarding feasibility of LESS surgery, it is technically challenging due to its systemic limitations, such as a crush between instruments, an unstable camera platform, the limited mobility of straight instruments, and the lack of instrument triangulation [5]. Due to these limitations, surgeon needs a sustained learning curve period to achieve the proficiency to perform the LESS surgery without technical difficulty. Intracorporeal suturing, in particular, is difficult with a steep learning curve when performed using standard laparoscopic needle drivers [6].

The technology and techniques related to robotic surgery are still evolving in the direction of easier minimal invasive surgery. Robotic surgery has greatly improved surgeon dexterity, surgical precision, visualization, ergonomics and allowed procedures that were performed by laparotomy to be performed by laparoscopy. However, robotic surgery has substantially increased the number and size of ports required compared with LESS [7, 8]. Therefore, the concept of combining LESS and robotic surgical systems seems to be a promising choice to overcome the technical complexities of the LESS and satisfied cosmetic result [9, 10]. If the robotic single-site (RSS) surgery has comparable surgical outcomes with LESS, we expect that RSS surgery will be an optimal surgical approach for benign gynecologic disease. The aim of this study was to compare the surgical outcomes of RSS (RSS-H) and LESS total hysterectomy (LESS-H) and to evaluate the feasibility of RSS-H in patients with benign gynecologic disease.

## Materials and methods

### Patients

**Fig. 1:**
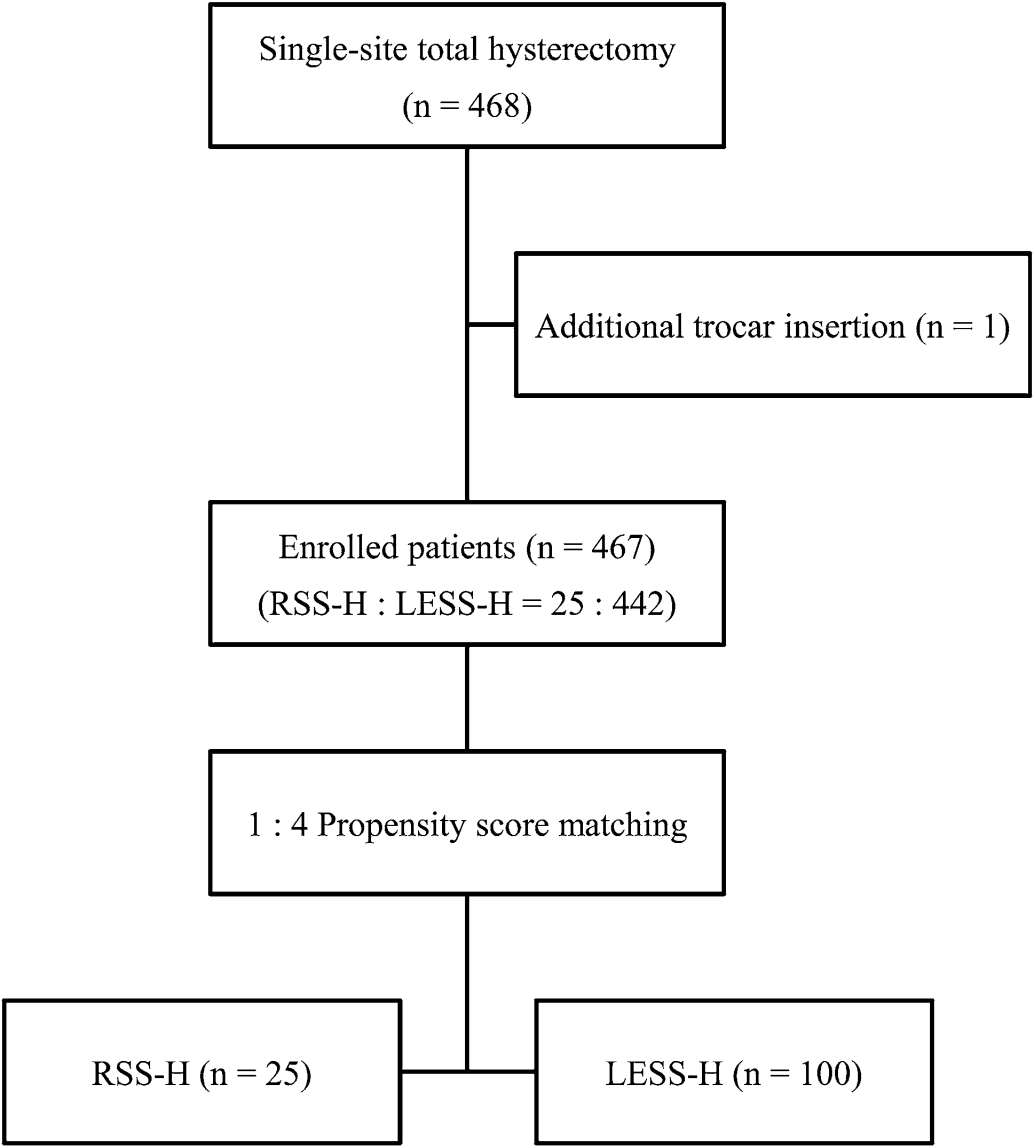
Flow diagram of the study. *RSS*-*H* robotic single-site hysterectomy; *LESS*-*H* laparoendoscopic single-site hysterectomy

Written informed consent for use of a new technique was obtained from all patients prior to surgery. Between March 2011 and December 2014, we identified 468 consecutive patients who underwent RSS-H or LESS-H for benign gynecologic disease at Ajou University Hospital. We performed LESS-H regardless of the size of the uterus in all patients who want to undergo laparoscopic surgery. Consequently, 443 patients underwent LESS-H for benign disease during the study period. We enrolled all patients to this analysis during this period. There was no case of multi-port laparoscopic hysterectomy. Robotic hysterectomy was performed using only RSS system since the RSS system has been introduced to our institute and all of cases were included in this analysis. Of these, one patient who needed additional trocars due to severe adhesion during LESS-H was excluded. Finally, we enrolled 467 patients in this study who were divided into two groups based on the approach of surgery (RSS-H group, *n* = 25, and LESS-H group, *n* = 442) (Fig. 1). In this analysis, all surgery was performed by a single surgeon (J. Paek) who had experiences of more than 250 cases of robotic surgery and 800 cases of LESS surgery for gynecologic disease. Patient status was estimated in terms of operating time, estimated blood loss (EBL), serum hemoglobin (Hb) drop (change between the preoperative Hb and the Hb 1 day after surgery), postoperative pain, length of postoperative hospital day (POD), and operative complications. Operating time was categorized as the time for hysterectomy until the intracorporeal detachment of the uterus, time for the closure of the vaginal cuff, and the total operating time. The total operating time was defined as time from the skin opening to the closure and included docking time, time for hysterectomy, time for the uterus removal, time for cuff closure, and time for incision site repair. Postoperative pain assessments were performed in all patients using a validated visual analog pain scale. The scale was presented as a score from 0 to 10, with verbal descriptors anchored with ‘no pain’ and ‘agonizing pain.’ Patients were asked to rate their pain intensity at 12, 24, and 48 h after surgery. In addition, we classified complications into minor and major complications. Minor complications included fever >38.5 °C more than 2 days after surgery and delay of discharge plan. Major complications included the situation requiring a secondary surgical procedure to perform adequate hemostasis and repair of urinary tract injuries or bowel perforation.

### Surgical techniques

**Fig. 2:**
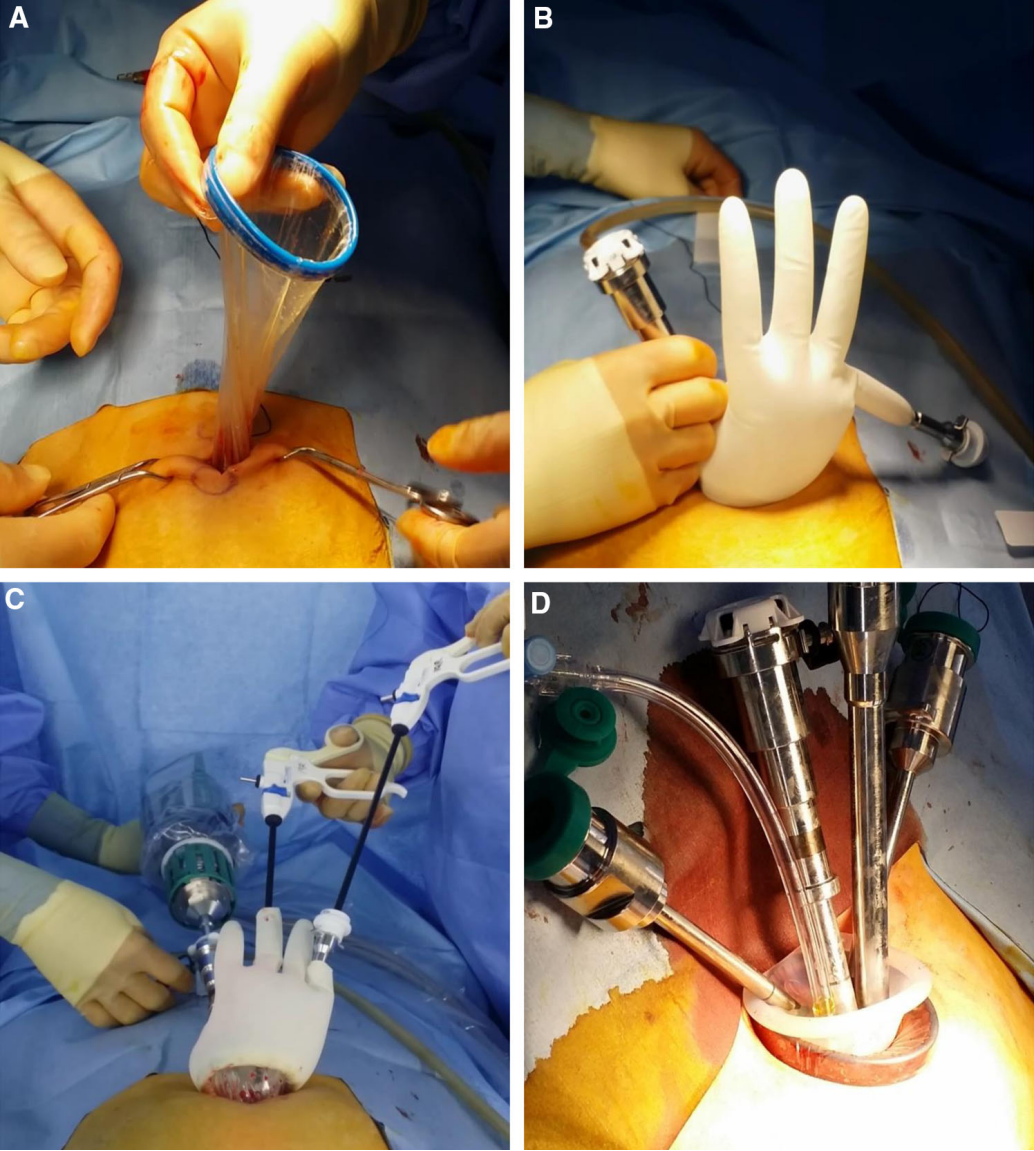
Port placement for RSS surgery. **A** Insertion of a wound retractor. **B** Making a single multi-channel port and inserting RSS cannulae. **C** Performing adhesiolysis using laparoscopic instruments. **D** Insertion of RSS platform and cannulae after removing surgical glove

For the RSS-H, the da Vinci single-site surgical platform (Intuitive Surgical, Inc., CA, USA) was used. After a 2.5-cm vertical skin incision was made in the umbilicus, the abdominal cavity was entered using the open technique. Before inserting the RSS platform, we made a single multi-channel port using a wound retractor and surgical glove and explored the pelvic cavity (Fig. 2A, B). If there was pelvic adhesion, adhesiolysis was performed using laparoscopic instruments before setting up robotic system (Fig. 2C). The RSS platform was inserted through a wound retractor after surgical glove was removed (Fig. 2D). The robot was docked between the patient’s legs. The RSS system incorporates a multi-channel port which accommodates 2 curved robotic cannulas and a 5- or 10-mm laparoscopic instrument. The instruments and accessories include crocodile grasper, monopolar hook cautery, Maryland bipolar cautery, and needle driver. For hysterectomy, the round ligaments were ligated bilaterally and bilateral infundibulopelvic or utero-ovarian ligaments were securely skeletonized and transected using an ENCEAL straight tissue sealer (Ethicon Endo-surgery, Ohio, USA) after identification of the ureters. The anterior and posterior leaves of the broad ligament were excised with a monopolar hook. The bladder and the attached peritoneal flap were developed using a monopolar hook. Both uterosacral ligaments were excised with the monopolar hook, and the peritoneum on the posterior cervix was excised and divided from the cervix. The anterior colpotomy and posterior colpotomy were performed with the monopolar hook in the vagina delineated with colpotomizer. Then, both uterine vessels were skeletonized and desiccated with an ENCEAL. Once the bladder was dissected below the colpotomy cup, circumferential colpotomy was performed using the monopolar hook. The resected uterus was extracted through the vagina. For the closure of vaginal cuff, we performed continuous running suture intracorporeally using a barbed suture.

For the LESS-H, a single multi-channel port system was used. Briefly, after making a 1.5-cm vertical intra-umbilical skin incision, a wound retractor was inserted into the peritoneal cavity through the umbilicus. A 7½ surgical glove was fixed to the outer ring of the wound retractor. After making small incisions in the fingertip portions of the glove, three 5-mm trocars were inserted. A rigid 30-degree, 5-mm endoscope was used. The procedure of hysterectomy was equal to that of RSS-H. No any drainage tube was inserted, and the umbilical fascia and subcutaneous tissue was approximated with 2-0 Vicryl sutures without a skin suture in both groups.

### Postoperative management

All patients were permitted sips of water starting 6 h after surgery. A soft diet was offered as the first meal after passing flatus, and then patients were offered a general diet. The intravenous pain control anesthesia (IV-PCA) was administered to the patients who wanted it before surgery. The patients were administered IV-PCA using fentanyl, with a basal infusion of 15 μg/h, bolus dose of 15 μg, and lockout interval of 15 min. Generally, the IV-PCA was used until POD 1 or 2 according to the frequency which patients push a button for administration of medication. If another pain control was needed, 30 mg ketorolac was administered intravenously. Urinary Foley catheters were removed on the morning of POD 1, and patients were encouraged to ambulate starting at POD 1. The patients were discharged from hospital at POD 3 unless they have postoperative complications, such as abdominal pain and fever.

### Statistical analysis

All continuous data are expressed as mean ± SD, and categorical data are reported as an absolute number or percentage. Frequency distributions were compared using Chi-square test, and mean or median values were compared using Student’s *t* and Mann–Whitney *U* tests. All calculated *p* values were two-sided, and *p* < 0.05 was considered statistically significant. Data were analyzed using SAS/STAT software, version 9.4 (SAS Institute Inc., NC, USA). To reduce the effect of selection bias and potential confounding in this retrospective cohort study, estimated propensity scores were used to match the RSS-H group to LESS-H group. In our study, this was computed for each of the patients using a logistic regression model including the following variables: age, body mass index, the presence of previous abdominal surgery, the presence of pelvic adhesion, and the size of the uterus. The propensity score model was well-calibrated (Hosmer–Lemeshow goodness-of-fit test, *p* = 0.6104) with good discrimination (c-statistic = 0.688). Based on the propensity scores, 25 patients who underwent RSS-H were matched to 100 patients who underwent LESS-H (Fig. 1).

## Results

All operations were completed robotically or laparoscopically with no additional port insertion or conversion to laparotomy. A summary of subject characteristics is described in Table 1. Compared with the LESS-H group, the RSS-H group had more previous abdominal surgery history (64 vs 37 %, *p* = 0.008) and more pelvic adhesion (48 vs 21 %, *p* = 0.002). Surgical outcomes are shown in Table 2. Compared to the LESS-H group, the RSS-H group had longer operating times (170.9 ± 65.5 vs 88.3 ± 38.4 min, *p* < 0.0001) and less operative bleeding (median EBL, 20 vs 50 ml, *p* = 0.040; mean Hb drop, 1.6 vs 2.0 g/dl, *p* = 0.031). For the LESS group, the rate of minor complications was 1.13 % (*n* = 5) and all of patients complaint abdominal pain with fever after POD 3. The reasons for symptoms were ileus and inflammation in the pelvic cavity. They were administered antibiotics intravenously and discharged after the symptoms improved completely. The rate of major complication was 1.36 % (*n* = 6). Of these, 2 patients (0.45 %) needed the insertion of percutaneous nephrostomy due to delayed ureter injuries and 2 patients underwent secondary laparoscopic surgery due to massive hemorrhage immediately after surgery. The hemoperitoneum was seen due to hemorrhage in the umbilicus. Another 2 patients suffered vault dehiscence right after sexual intercourse at 2 months after surgery and underwent laparoscopic vault repair. For the RSS-H group, there was no perioperative complication. After one-to-four propensity score matching was performed, the RSS-H group still showed longer operating times (170.9 ± 65.5 vs 94.1 ± 44.3 min, *p* < 0.0001) and less operative bleeding (median EBL, 20 vs 50 ml, *p* = 0.009; mean Hb drop, 1.6 vs 2.0 g/dl, *p* = 0.038) compared to the LESS-H group. To remove the effects of the use of IV-PCA on the postoperative pain, we divided the both groups into IV-PCA and IV-PCA naïve group. As a result, the RSS-H group showed less postoperative pain 12 h after surgery than the LESS-H and the median number of painkillers given was the same in both groups (Table 3). Abdominal wounds cleanly healed in all patients without any complication.

**Table 1:** Patient demographics

	Overall series			Propensity score–matched pairs		
	RSS-H (*n* = 25)	LESS-H (*n* = 442)	*p* value	RSS-H (*n* = 25)	LESS-H (*n* = 100)	*p* value
Age (years)	48.0 ± 4.1	48.9 ± 8.7	0.382	48.0 ± 4.1	48.1 ± 7.6	0.979
BMI (kg/m^2^)	24.3 ± 2.5	24.0 ± 3.3	0.608	24.3 ± 2.5	24.1 ± 2.6	0.690
Previous abdominal surgery			0.008			0.783
No	9 (36 %)	277 (62.7 %)		9 (36 %)	39 (39 %)	
Yes	16 (64 %)	165 (37.3 %)		16 (64 %)	61 (61 %)	
Pelvic adhesions			0.002			0.788
No	13 (52 %)	350 (79.2 %)		13 (52 %)	49 (49 %)	
Yes	12 (48 %)	92 (20.8 %)		12 (48 %)	51 (51 %)	
Weight of uterus (g)	271 ± 119	249 ± 190	0.386	271 ± 119	294 ± 210	0.471
Histology						
Leiomyoma	16 (64 %)	230 (52 %)				
Adenomyosis	3 (12 %)	73 (16.5 %)				
CIN	2 (8 %)	81 (18.3 %)				
Endometrial hyperplasia	4 (16 %)	29 (6.6 %)				
Uterine prolapse		23 (5.2 %)				
Hydatidiform mole		6 (1.4 %)				

*RSS*-*H* robotic single-site total hysterectomy, *LESS*-*H* laparoendoscopic single-site total hysterectomy, *BMI* body mass index, *CIN* cervical intraepithelial neoplasia

**Table 2:** Operative outcomes

	Overall series			Propensity score–matched pairs		
	RSS-H (*n* = 25)	LESS-H (*n* = 442)	*p* value	RSS-H (*n* = 25)	LESS-H (*n* = 100)	*p* value
Mean operating time (min)						
Time for hysterectomy	81.8 ± 49.6	52.2 ± 27.7	0.007	81.8 ± 49.6	56.3 ± 31.9	0.021
Time for cuff closure	17.8 ± 10.8	15.0 ± 3.3	0.202	17.8 ± 10.8	14.5 ± 2.7	0.141
Total operating time	170.9 ± 65.5	88.3 ± 38.4	<0.0001	170.9 ± 65.5	94.1 ± 44.3	<0.0001
(Docking time)	14.0 ± 4.7			14.0 ± 4.7		
(Console time)	99.6 ± 49.7			99.6 ± 49.7		
Median estimated blood loss (ml, IQR)	20 (30)	50 (30)	0.040	20 (30)	50 (30)	0.009
Mean serum hemoglobin drop (g/dl)	1.6 ± 1.0	2.0 ± 0.9	0.031	1.6 ± 1.0	2.0 ± 0.9	0.038
Transfusion requirement	0	3 (0.68 %)		0	0	
Mean POD (days)	3.5 ± 0.7	3.8 ± 1.4	0.111	3.5 ± 0.7	3.8 ± 1.4	0.429
Perioperative complications						
Major	0	6 (1.36 %)		0	2 (2 %)	
Minor	0	5 (1.13 %)		0	2 (2 %)	

*RSS*-*H* robotic single-site total hysterectomy, *LESS*-*H* laparoendoscopic single-site total hysterectomy, *IQR* interquartile range, *POD* postoperative hospital days

**Table 3:** Postoperative pain

	Propensity score–matched pairs					
	IV-PCA naive			IV-PCA		
	RSS-H (*n* = 11)	LESS-H (*n* = 74)	*p* value	RSS-H (*n* = 14)	LESS-H (*n* = 26)	*p* value
Median postoperative pain score (range, IQR)						
12 h	3 (2–4, 0)	4 (2–5, 1)	<0.0001	3 (3–4, 1)	4 (3–4, 1)	0.028
24 h	3 (1–3, 1)	3 (2–4, 0)	0.150	3 (2–4, 0)	3 (2–3, 1)	0.035
48 h	3 (1–3, 1)	3 (1–4, 1)	0.563	3 (1–3, 1)	3 (1–4, 1)	0.670
Median number of painkillers given (range, IQR)	1 (0–2, 0)	1 (0–3, 0)	0.594	1 (0–2, 0)	1 (0–2, 0)	0.680

*IV*-*PCA* intravenous patient controlled analgesia, *RSS*-*H* robotic single-site total hysterectomy, *LESS*-*H* laparoendoscopic single-site total hysterectomy, *IQR* interquartile range

## Discussion

The RSS system, as it is known, is originally developed for cholecystectomy [11]. Unlike RSS cholecystectomy, gynecologic surgeons can have trouble controlling the RSS instruments because surgical targets are so hard or huge in gynecologic disease. However, several reports showed that RSS-H was feasible and safe and allowed for optimal postoperative pain control and improved cosmetic results with advantages over laparoscopic surgery of superiority of magnified surgical view and more precise dissection [12–15]. In addition, Bogliolo et al. [15] showed that RSS-H could be reproducible procedure with comparison of similar operative outcomes between two institutions.

**Fig. 3:**
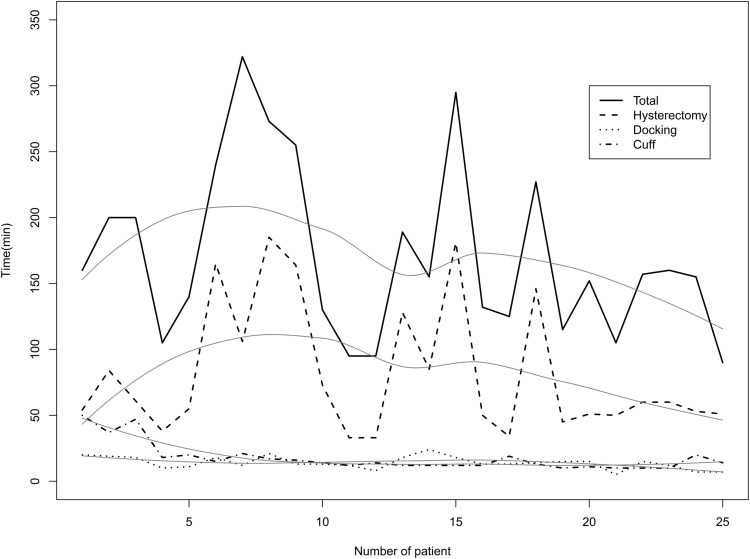
Raw operating time data in the RSS-H with linear (*black*) and quadratic (*red*) *trend lines* (Color figure online)

In this study, we aimed to appropriately perform the RSS surgery and to surmount technical problems of LESS at the same time. To reduce the effect of selection bias and potential confounding in this retrospective cohort study, we used estimated propensity scores that match the RSS-H group to LESS-H group. Although we have no specific indication for RSS-H, we could expect that the used propensity score model was well-calibrated by adjusting variables. As a result, the RSS-H group showed longer operating times, less operative bleeding, and less postoperative pain immediately after surgery compared to the LESS-H group. Although our result had longer total operating times than that of previous studies, there was no difference in docking or console time [15, 16]. In other words, there was difference of times for removal of the uterus, not actual operating times. Additionally, the size of the uterus of patients enrolled in our study was huge compared to other studies. The raw operating time data of the RSS-H are shown in Fig. 3. The analyzed patients are too small to speculate the proficiency of the RSS-H. However, it seems to reach the proficiency more or less if it is considered that the time for uterus removal through the vagina was long (>100 min) in all of 5 patients who showed long total operating time (>240 min). At our early experience of RSS-H, it took a long time, over 40 min, to perform cuff closure. However, we could achieve proficiency of cuff closure after 5 cases and there was no difference of the time for cuff closure compared to the LESS-H (13.7 vs 14.5 min, *p* = 0.243). Because the needle driver of RSS is non-articulated and semi-rigid, we need a cutting and straight needle. It was difficult for operator to handle huge and firm fibroids using semi-rigid instruments of the RSS-H, and it induced longer operating times compared to LESS-H. In addition, we made a single multi-channel port using a surgical glove and checked the presence of adhesion before the docking of the RSS system. And, we had to repeat un-docking and re-docking process before and after removing the uterus. These steps are induced by the technical or structural characteristics of the RSS system and are included in total operating time. Although we did not show the time for these steps in this study separately, it took about 20 min. Although this step needs additional operating time, this can be a useful technical tip. It is so difficult to perform adhesiolysis between the omentum or bowel and peritoneum because the RSS cannula is close to the adhesion site. Therefore, we can perform adhesiolysis using the single multi-channel port and laparoscopic instruments before docking the RSS system. In addition, a wound retractor that used to make the multi-channel port causes the abdominal wall to be thin and helps the RSS platform not detach from obese patients.

**Fig. 4:**
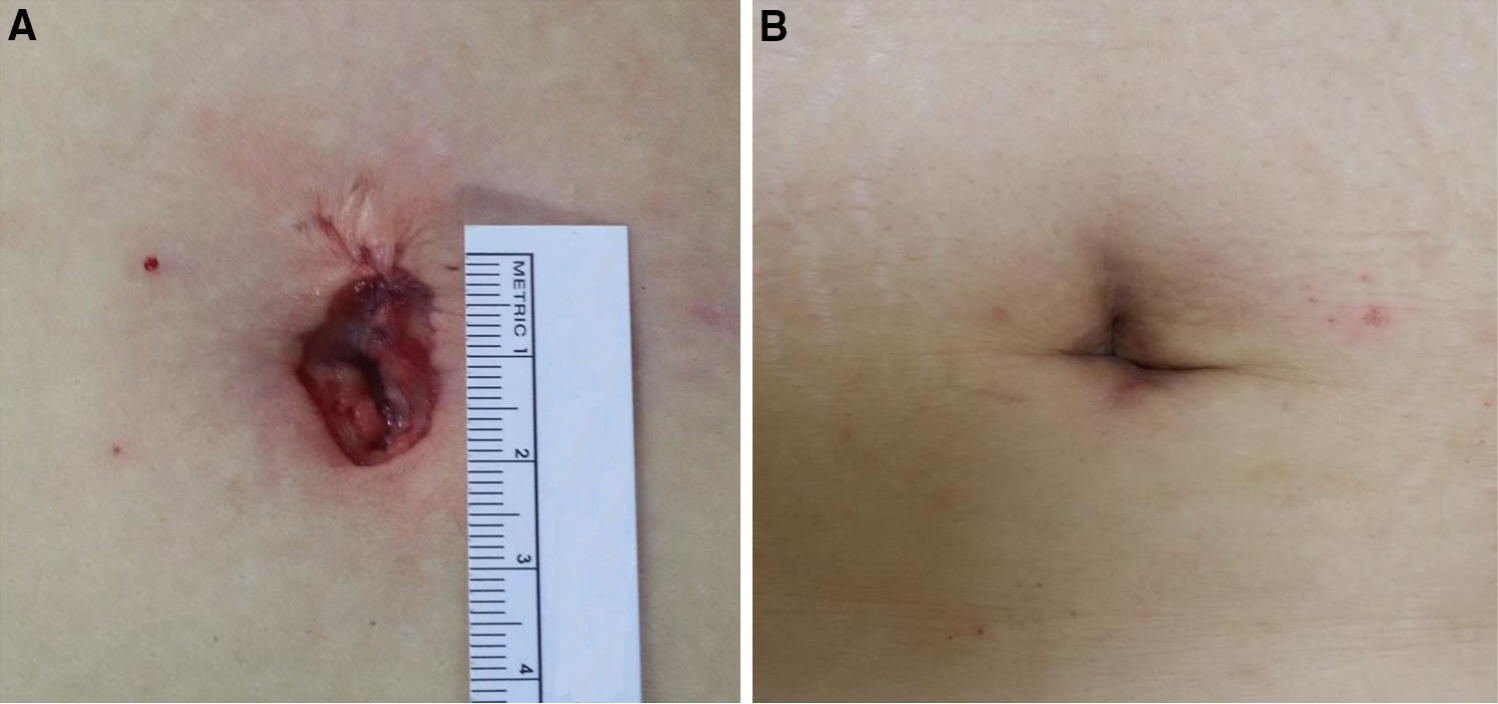
Postoperative photograph of skin incision. **A** View immediately after surgery. **B** View after 6 weeks postoperatively

For pain assessments, it seems to be unreasonable to speculate that RSS-H shows less postoperative pain than LESS-H with the results of pain score only at 12 h after surgery. However, the RSS instrument can function without excessive movement because the RSS platform is fixed completely to the umbilicus. On the other hand, for the LESS, laparoscopic instruments move tightly because we make small incision of 1.5 cm. We expect that it may reduce postoperative pain not to stretch the incision site during surgery. As a result, the median pain score measured at 12, 24, and 48 h after RSS-H did not exceed 3 in the visual analog pain scale and we could regard this score as a favorable result in our experience. The RSS surgery needs bigger skin incision than LESS surgery because we have to insert the platform for RSS system. However, the scar of RSS shrank and was hidden inside the umbilicus as times went on (Fig. 4). We could not see any scar outside the umbilicus at 6 weeks after surgery and expect that the RSS surgery have a comparable cosmetic outcome with LESS.

Regarding the technical limitations of RSS surgery, the current available RSS instruments have some differences from the conventional robotic wristed instruments. The procedure using non-articulated RSS instruments causes intracorporeal sutures, control of huge tumors and the uterus to be challenging. Secondly, the shaft of the RSS instrument is semi-rigid and does not have enough power to maintain traction of hard or heavy mass. Thirdly, the RSS system has long curved cannulae to compensate the weak strength of curved semi-rigid instruments. Because these long cannulae do not allow robotic arms to move toward the umbilicus freely, surgeons can feel technical difficulty toward enlarged mass or the uterus. Finally, the width of the jaw of the bipolar cautery is too narrow to use for the desiccation of the utero-ovarian ligament or infundibulopelvic ligament. Therefore, we used one of advanced bipolar devices to perform desiccation of the large vessels instead of the Maryland bipolar of the RSS. It is expected to save the operating time and avoid unnecessary thermal injury which can happen during the procedure of coagulation. Although there still remain technical limitations, these problems can be overcome as soon as advanced RSS instruments are developed; including wristed instruments, shorter cannulae, or fenestrated bipolar devices, are introduced.

## Conclusions

In conclusion, the RSS surgery is feasible and safe in selected patients with only minimal skin incisions and further experience and technical refinements will continue to improve operative results. In addition, prospective randomized trials will permit the evaluation of the potential benefits of the RSS surgery as a minimally invasive surgical approach.
